# Protective effect of microbisporicin (NAI-107) against vancomycin resistant *Enterococcus faecium* infection in a *Galleria mellonella* model

**DOI:** 10.1038/s41598-024-55262-8

**Published:** 2024-02-27

**Authors:** Nele Hofkens, Zina Gestels, Saïd Abdellati, Philippe Gabant, Hector Rodriguez-Villalobos, Anandi Martin, Chris Kenyon, Sheeba Santhini Manoharan-Basil

**Affiliations:** 1 Department of Clinical Sciences, Institute of Tropical Medicine Antwerp, 2000 Antwerp, Belgium; 2 Clinical Reference Laboratory, Department of Clinical Sciences, Institute of Tropical Medicine, 2000 Antwerp, Belgium; 3 Syngulon, Seraing, Belgium; 4 Cliniques Universitaires Saint-Luc, Avenue Hippocrate 10, 1200 Brussels, Belgium; 5 Department of Medicine, University of Cape Town, Cape Town, 7700 South Africa

**Keywords:** *Enterococcus faecium*, VanB, Lantibiotics, NAI-107, Microbisporicin, *Galleria mellonella*, Bacteria, Bacterial secretion

## Abstract

Increasing antimicrobial resistance in *Enterococcus faecium* necessitates the search for novel treatment agents, such as bacteriocins. In this study, we conducted an in vivo assessment of five bacteriocins, namely Lacticin Z, Lacticin Q, Garvicin KS (ABC), Aureocin A53 and Microbisporicin (NAI-107), against vanB-resistant *Enterococcus faecium* using a *Galleria mellonella* model. Our in vitro experiments demonstrated the efficacy of all five bacteriocins against vanB-resistant *E. faecium* with only NAI-107 demonstrating in vivo efficacy. Notably, NAI-107 exhibited efficacy across a range of tested doses, with the highest efficacy observed at a concentration of 16 µg/mL. Mortality rates in the group treated with 16 µg/mL NAI-107 were lower than those observed in the linezolid-treated group. These findings strongly suggest that NAI-107 holds promise as a potential alternative therapeutic agent for treating infections caused by resistant *E. faecium* and warrants further investigation.

## Introduction

*Enterococcus faecium and Enterococcus faecalis* are Gram-positive facultative anaerobes that are commonly found in the gastrointestinal tracts of humans and animals^1,2^. Infections caused by vancomycin-resistant enterococci (VRE) emerged in Europe in the 1980’s and are becoming an increasing problem worldwide^1,3^. The vast majority of VRE infections are caused by *E. faecium*. Surveillance data from the United States revealed that the prevalence of vancomycin resistance in *E. faecalis* and *E. faecium* was 7% and 80%, respectively^4^.

Typically, resistance to vancomycin in enterococci is due to the presence of the *vanA* or *vanB* genes^5^. They are situated on a transposon (Tn1546 for *vanA*, Tn1547, or Tn5382 for *vanB*)^5^. The *vanA* gene results in a high level of resistance to both vancomycin and teicoplanin, while *vanB* exclusively provides resistance to vancomycin^6,7^. Vancomycin-resistant *E. faecium* infections are particularly difficult to treat, given their resistance to beta-lactams^1,8^. Treatment options include linezolid, but combined linezolid-vancomycin resistance is possible^9^. Linezolid toxicity is a frequent problem, and the utility of this agent for certain types of infection, such as endocarditis, is uncertain^8–10^.

This paucity of treatment options for VRE infections provided the motivation for this study, where we aimed to assess if bacteriocins that show in vitro activity against VRE would exhibit this activity in vivo. Bacteriocins have gained considerable attention as potential alternatives to traditional antibiotics due to their narrow-spectrum activity and a lower likelihood of inducing resistance^11,12^. Most leaderless bacteriocins such as Lacticin Q (53 aa), Lacticin Z (53 aa), and Garvicin KS have proven to have inhibitory effects against a range of genera such as *Listeria, Staphylococcus* and *Enterococcus*^13,14^. The lantibiotic NAI-107 has been found to be active against a wide range of multi-drug resistant (MDR) Gram-positive bacterial pathogens, including methicillin resistant *Staphylococcus aureus* (MRSA), glycopeptide-intermediate resistant *S. aureus* (GISA) and vancomycin-resistant Enterococci (VRE)^12,15,16^. Additionally, NAI-107 was found to be effective against vanA *E. faecium* 569 and vanA *E. faecalis* A533 in vivo when administered intravenously to neutropenic mice^17^. In addition to bacteriocins, natural antimicrobial compounds or semi-synthetic derivatives of microbial natural products such as *Nigella sativa* oil extract, bacillusin A (23), Nicrophorusamide A**,** Urnucratin A have been shown to exhibit promising efficacy against VRE^18–20^.

*G. mellonella* has been shown to be an effective model host for investigating the efficacy of antimicrobial agents against various pathogens^21,22^. This model serves as a viable alternative to traditional mammalian in vivo systems. The immune response in *Galleria mellonella* encompasses both cellular mechanisms—such as phagocytosis—and humoral responses, which include melanization, hemolymph clotting, and the synthesis of antimicrobial peptides^23–25^. Crucially, studies have found that the efficacies of antimicrobial agents on infected *G. mellonella* larvae closely correlate with the known drug susceptibilities of the pathogens in vitro and in mammalian models^26–29^. In addition, the use of *G. mellonella* enables the application of the 3Rs principles (replacement, reduction and refinement) in animal experimentation^29^.

We therefore explored a panel of bacteriocins from PARAGEN collection (Syngulon) and Microbisporicin NAI-107 (Naicons, SRL) against vanB-resistant *E. faecium* in a *G. mellonella* model. The PARAGEN collection consists of 66 bacteriocins and four of these bacteriocins (Lacticin Z, Lacticin Q, Garvicin KS (ABC), Aureocin A53) showed in vitro antimicrobial activity against vanB-resistant *E. faecium* (Table 1)^30^.

**Table 1:** The MICs of the antibiotics (n = 8) and five most promising bacteriocins were determined using the micro broth dilution method for van-B type *E. faecium* (ID 553).

	MIC (mg/L)	MIC (µM/L)
*Antibiotics*		
Ampicillin	≥ 32	–
Vancomycin	64	–
Linezolid	2	–
Gentamicin	≥ 32	–
Tetracycline	0.25	–
Ciprofloxacin	≥ 4	–
Erythromycin	≥ 8	–
Teicoplanin	0.5	–
*Bacteriocins*		
Lacticin Q	6.24	1.05763
Lacticin Z	1.56	0.26263
Aureocin A53	1.56	0.26087
Garvicin KS (ABC)	0.78	0.08058
Microbisporicin	4	0.00178

## Materials and methods

### *E. faecium* strains and minimum inhibitory concentrations (MIC) of bacteriocins and antimicrobial drugs

VanB-type *E. faecium* (ID 553) was used for all the experiments. Sixty-six bacteriocins from Syngulon PARAGEN collection and NAI-107 from Naicons, SRL were screened using the resazurin microtiter assay (REMA)^30,31^. The MIC for the antimicrobials listed in Table 1 were determined according to the clinical and laboratory standard institute (CLSI) guidelines and the European Committee on Antimicrobial Susceptibility Testing (EUCAST) broth microdilution reference method for MIC determination in *E. faecium*^32,33^.

### In vivo studies

- Determining the appropriate dose of *E. faecium*

*G. mellonella* larvae were treated with a range of doses of vanB-type *E. faecium* (ID 553) (1 × 10^6^–1 × 10^9^ CFU/mL) and observed for death alongside phosphate buffer saline (PBS) controls. The dose that resulted in approximately 80% mortality was 2.7 × 10^7^ CFU in 30 µl PBS, which was further used in the *G. mellonella* infection model.

(b) *Galleria mellonella* infection model

Injection of *G. mellonella* was carried out as described in Dijokaite et al.^21^. The last larval stage of *G. mellonella* (Terramania, Arnhem, NL) were used for the experiments. Only non-discoloured, healthy larvae were selected. The larvae were injected in the last left proleg using 0.3 mL U-100 insulin syringes (BD Micro-Fine).

Each test group consisted of two groups of 10 larvae per concentration of each bacteriocin or linezolid (10 µg/mL). 30 µl of PBS containing 2.7 × 10^7^ CFU *E. faecium* was used to infect the *G. mellonella* larva. The larvae were treated with various concentrations of bacteriocins. One positive and two negative control groups were used. The positive control group received only the *E. faecium* inoculum in PBS. Out of the two negative control groups, one group underwent no manipulation, while the other group was injected with PBS only. Each antimicrobial agent was tested in a single experiment using larvae from the same batch and tested at the same time under identical conditions. Each group were incubated in sterile Petri dishes at 37 °C with a 5% (v/v) CO_2_ atmosphere for the length of the experiments. The larvae were observed for 120 h for any indications of illness, necrosis, or paralysis, which enabled an evaluation of the bacteriocin's toxicity. Larvae were scored dead if they did not respond to touch stimuli by blunt sterile forceps and scored for 5 consecutive days (120 h).

**Figure 1:**
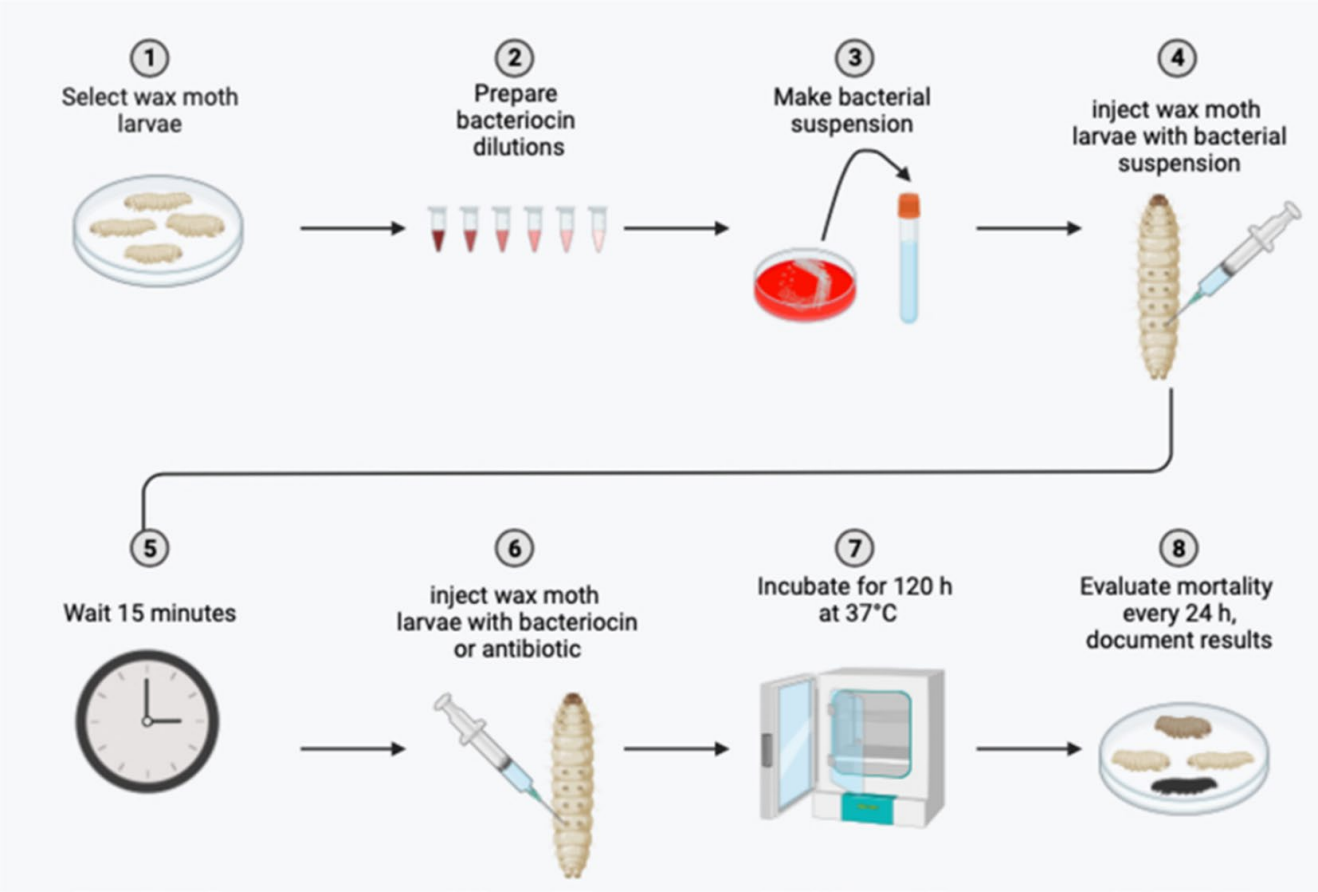
Overview of the in vivo assay in *Galleria mellonella* model. (Figure was generated using BioRender).

An overview of the in vivo assay in *Galleria mellonella* model is shown in Fig. 1.

### Statistical analysis

Data were analyzed using GraphPad Prism v9. Survival plots were created using Kaplan–Meier survival curves. Statistical analysis was carried out using the Mantel-Cox test to compare survival curves between the PBS controls and each treatment arm. A *P* value of < 0.05 was considered statistically significant.

## Results

### MIC determination

Five of the bacteriocins, Lacticin Q (LcnQ), LacticinZ (LcnZ), Aureocin A53 (AucA), Garvicin KS (ABC) and microbisporicin (NAI-107) had the highest antimicrobial activity against van-B type *E. faecium* and were selected for the in vivo experiments (Table 1, Supplementary Table S1). The MICs of the antimicrobials and bacteriocins are listed in Table 1.

### In vivo assay for efficacy of bacteriocins in *Galleria mellonella E. faecium* infection model

**Figure 2:**
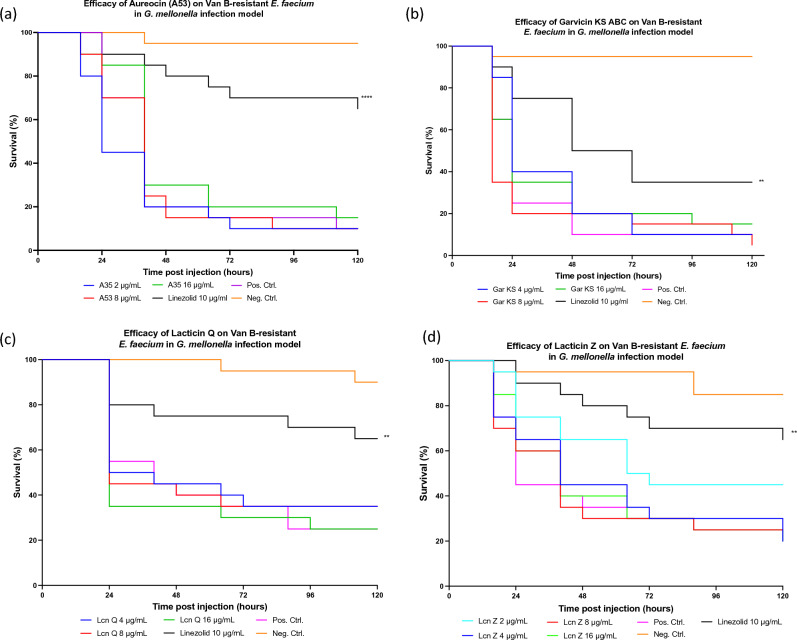
Efficacy of (**a**) Aureocin A53 (2, 8, and 16 µg/mL) and Linezolid (10 µg/mL) (**b**) Garvicin KS ABC (4, 8, and 16 µg/mL) (**c**) Lacticin Q (4, 8, and 16 µg/mL) and (**d**) Lacticin Z (2, 4, 8, and 16 µg/mL) treatment on *G. mellonella* larvae. A Negative control group was injected with 30 µL of PBS, and a positive control group was inoculated with 30 µL of PBS containing 2.7 × 10^7^ CFUs of the *E. faecium* strain. Both test and control groups consisted of 20 larvae and were incubated for 120 h at 37 °C. Symbols represent mean survival of the *G. mellonella*. *P < 0.01, **P < 0.001, ***P < 0.0001, ****P < 0.00001.

The in vivo efficacy of A53, GarKS (ABC), lacticin Q, lacticin Z and NAI-107 were determined against vanB-type *E. faecium* in comparison to linezolid. Despite the in vitro activity of all five of these bacteriocins*,* four of the bacteriocins [A53, GarKS (ABC), lacticin Q and lacticin Z) did not demonstrate any detectable efficacy in vivo (Fig. 2).

**Figure 3:**
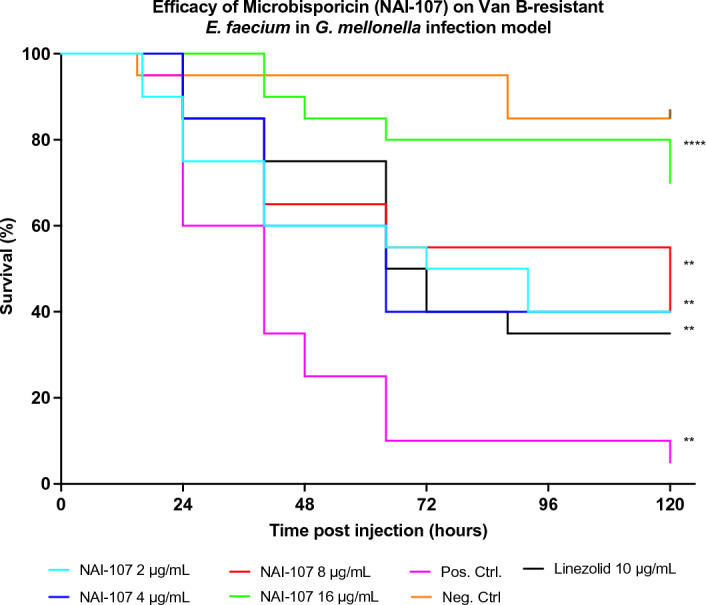
Effectiveness of Microbisporicin (2, 4, 8 and 16 µg/mL) and Linezolid (10 µg/mL) treatments on *G. mellonella* larvae. A negative control group was injected with 30 µL of PBS, and a positive control group was inoculated with 30 µL of PBS containing 2.7 × 10^7^ CFUs of the *E. faecium* strain. Both test and control groups consisted of 20 larvae and were incubated for 120 h at 37 °C. Symbols represent mean survival of the *G. mellonella*. *P < 0.01, **P < 0.001, ***P < 0.0001, ****P < 0.00001.

NAI-107 was effective in vivo at all doses tested—2 µg/mL, 4 µg/mL, 8 µg/mL 16 µg/mL (*P* values 0.01 to < 0.0001; Fig. 3). Mortality was lowest in the 8 µg/mL and 16 µg/mL groups. The mortality in the 16 µg/mL was significantly lower than the linezolid treated group (*P* = 0.0221).

## Discussion

In our study, we observed that among the bacteriocins investigated, only NAI-107 demonstrated effectiveness in protecting *Galleria mellonella* from infection caused by vanB-type *Enterococcus faecium*. We do not have a definitive explanation for this difference, but several pharmacodynamic and pharmacokinetic factors may play a role. One of these is that all the bacteriocins, except NAI-107 may be metabolized by the *G. mellonella*^29,34^. Another possible explanation is that the mechanisms of action of these bacteriocins may differ. For example, the leaderless peptides such as Lacticin Q, typically form stable pores in bacterial membranes. In contrast, NAI-107 which are ribosomally synthesized peptides that undergo posttranslational modifications causes depolarization of the membrane resulting in bacterial cell death without the formation of stable pores^35,36^.

NAI-107 is a 23-amino acid lantibiotic (class I bacteriocin) produced by the actinomycete *Microbispora* sp. ATCC PTA-5024^12,35^. Like other lantibiotics, it contains a methyllanthionine and three lanthionine bridges^37^. Unlike the other lantibiotics, it also contains two unusually modified amino acids: 5-chlorotryptophan and 3,4-dihydroxyproline^37^. It has novel mechanisms of action, which include the inhibition of lipid II mediated synthesis of peptidoglycan as well as disrupting inner cell membrane protein interactions leading to slow membrane depolarization (Supplementary Fig. S1)^37^. Importantly, NAI-107 exhibits no known cross resistance with other antimicrobials^35^. Previous studies have revealed a low risk for the emergence of resistance during therapy^12^. One study found that after 20 subpassages at increasing concentrations of NAI-107, NAI-107 MICs of *N. gonorrhoeae, E. faecalis* (vanA), *E. faecium* (vanA) and *S. aureus* (MRSA and GISA) increased only occasionally and to a maximum of 2 to fourfold the initial MIC^12^. Thus far the predominant resistance mechanism to lantibiotics detected, has been the production of an immunity protein by the producer strain^12,35^. Its novel mechanism of action is a likely explanation for the fact that no cross resistance between NAI-107 and other antimicrobials has been found^35^.

NAI-107 is not absorbed orally, but it does have good bioavailability after intramuscular, intravenous and subcutaneous administration^1,38^. In a mouse model, single subcutaneous dosing of 5, 20 and 80 mg/kg resulted in Cmax concentrations from 4 to 22 µg/ml, AUC values from 27 to 276 mg h/Liter, and elimination half-lives of 4.2–8.2 h^39^. This characteristic implies that NAI-107 would require parenteral administration in multiple doses per day for therapeutic use.

There are a number of study limitations. We only evaluated the effects of the bacteriocins on a single strain of vanB-type *E. faecium* and did not include vancomycin-sensitive *E. faecium* (VSE) strains as a control. The hypothesis we were testing in this study was that NAI-107 would exhibit activity in vivo against VRE. This hypothesis was motivated by the growing problem of VRE infections. We acknowledge the limitations of this hypothesis. Inclusion of VSE strains in our hypothesis would have allowed us to assess if NAI-107 shows promise for both VSE and VRE infections. This would be of clinical use as the vancomycin susceptibility of clinical isolates is not typically known when they are first identified. Further studies will be required to assess if NAI-107 is active against VSE strains. Our findings need to be replicated in a broader range of strains of *E. faecium*. As already noted, we did not investigate the reasons why four of the five bacteriocins were active in vitro but not in vivo. We acknowledge that there is considerable variation in the therapeutic efficacy of linezolid between the different experiments testing each antimicrobial compound. This may be explained by differences in the larvae used in the different experiments. Each compound was assessed in a single experiment conducted with the same batch of larvae but each subsequent experiments was conducted with a new batch of larvae. This makes comparisons between experiments inappropriate. Our results may be reproduced using different infection vertebrate and invertebrate models, such as those involving mice, rats, rabbits, dogs, zebrafish, *Caenorhabditis elegans* and* Drosophila melanogaster* (reviewed in^40^). However, *Galleria mellonella* has been successfully used to model various aspects of the colonization and infection of *Enterococcus faecium*^40^. Finally, we did not assess the toxicity of the bacteriocins as this has been done in previous studies^27^. NAI-107 exhibited no toxicity up to 64 µg/mL in a *G. mellonella* model^27^. Notably, NAI-107 has been found to be non-toxic and efficacious against methicillin-resistant *Staphylococcus aureus* in a rat model^38^.

If future studies confirm that NAI-107 is non-toxic and efficacious in human trials, it may emerge as a useful option for treating multi-resistant enterococcal infections, addressing a critical need in the field of antimicrobial therapy.

### Supplementary Information

Supplementary Information.

## Data Availability

All data generated or analysed during this study are included in this published article. Additional information can be addressed to the corresponding author upon reasonable request.
