# Supplementary material for: Protective effect of microbisporicin (NAI-107) against vancomycin resistant Enterococcus faecium infection in a Galleria mellonella model
Source: Sci Rep. 2024 Feb 27;14:4786. doi: 10.1038/s41598-024-55262-8 (PMC10899196; doi:10.1038/s41598-024-55262-8)
Supplement: Supplementary file 1 — Supplementary Information. [file 41598_2024_55262_MOESM1_ESM.pdf]

Protective effect of microbisporicin (NAI-107) against vancomycin resistant *Enterococcus faecium* infection in a *Galleria mellonella* model

Nele Hofkens[1], Zina Gestels[1], Saïd Abdellati[2], Philippe Gabant[3], Hector Rodriguez-Villalobos[4], Anandi Martin[3], Chris Kenyon [1,5#], Sheeba Santhini Manoharan-Basil[1#*]

Supplementary Table 1. Bacteriocins from PARAGEN collection and sequences used in this study

Bacteriocin	Abbreviation	Producer organism	Length (aa)	Amino acid Sequence	Molecular weight (kda)
**Garvicin KS - A**	GarKS (ABC)	*Lactococcus garvieae*	34	MGAIIKAGAKIVGKGVLGGGASWLGWNVGEKIWK	3.45
**Garvicin KS - C**		*Lactococcus garvieae*	32	MGAIIKAGAKIVGKGALTGGGVWLAEKLFGGK	3.1
**Garvicin KS - B**		*Lactococcus garvieae*	34	MGAIIKAGAKIIGKGLLGGAAGGATYGGLKKIFG	3.16
**Lacticin Z**	LcnZ	*Lactococcus lactis*	53	MAGFLKVVQILAKYGSKAVQWAWANKGKILDWINAGQAIDWVVEKIKQILGIK	5.94
**Lacticin Q**	LcnQ	*Lactococcus lactis*	53	MAGFLKVVQLLAKYGSKAVQWAWANKGKILDWLNAGQAIDWVVSKIKQILGIK	5.9
**Aureocin A53**	AucA	*Staphylococcus aureus*	51	MSWLNFLKYIAKYGKKAVSAAWKYKGKVLEWLNVGPTLEWVWQKLKKIAGL	5.98

The molecular weight for the bacteriocins were deduced using https://www.bioinformatics.org/sms/prot_mw.html

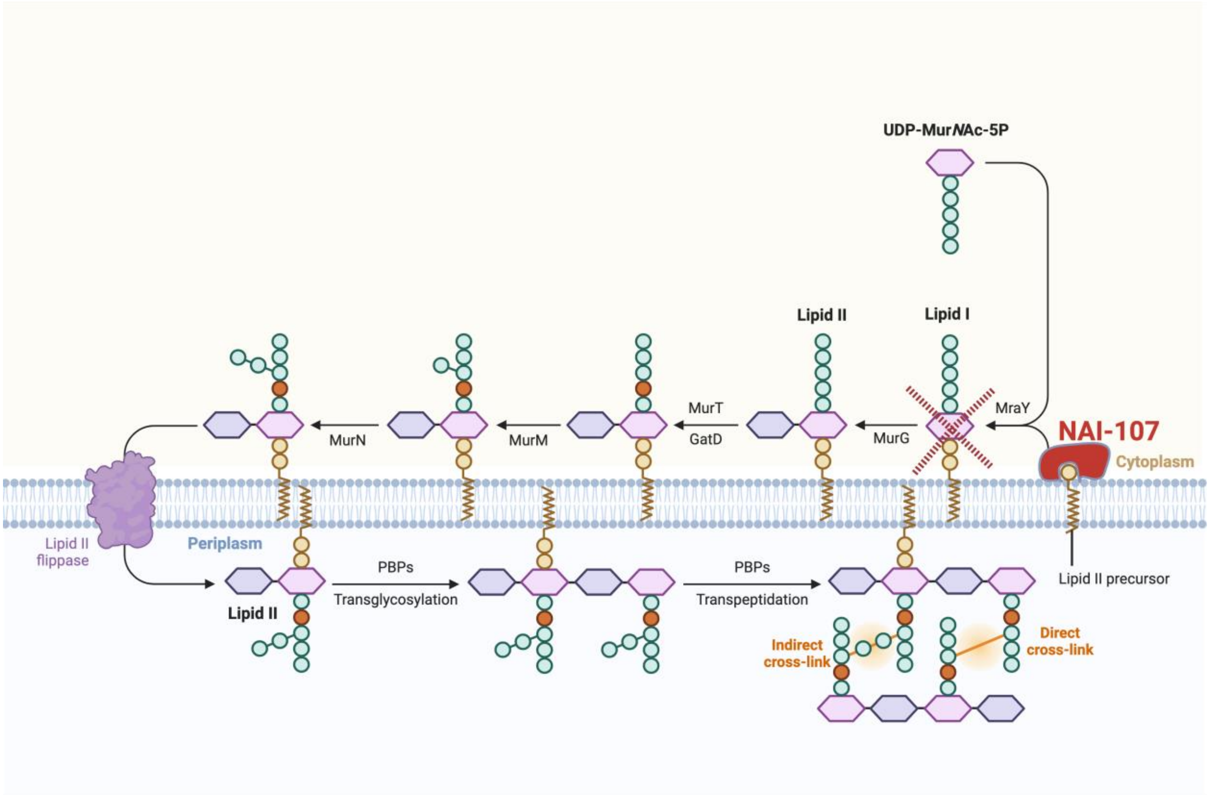

Supplementary Figure 1. Schematic figure of the peptidoglycan biosynthesis and how it is thought to be inhibited by NAI-107. NAI-107 (red-coloured figure) binds to the lipid II precursor (yellow-coloured figure) and inhibits the formation of lipid I (red cross), resulting in the inhibition of the further pathway of the peptidoglycan biosynthesis. (Figure made in BioRender based on literature review [1-3] and adapted from [4]. This image is distributed under the terms of the Creative Commons Attribution License (CC-BY), a copy of which is available at https://creativecommons.org/licenses/by/4.0/).

References

1. Brunati, C., et al., *Expanding the potential of NAI-107 for treating serious ESKAPE pathogens: synergistic combinations against Gram-negatives and bactericidal activity against non-dividing cells.* Journal of Antimicrobial Chemotherapy, 2018. **73**(2): p. 414-424.
2. Münch, D., et al., *The lantibiotic NAI-107 binds to bactoprenol-bound cell wall precursors and impairs membrane functions.* Journal of Biological Chemistry, 2014. **289**(17): p. 12063-12076.
3. Cruz, J.o.C., et al., *Brominated variant of the lantibiotic NAI-107 with enhanced antibacterial potency.* Journal of natural products, 2015. **78**(11): p. 2642-2647.
4. Zhou, J., et al., *Breaking down the cell wall: Still an attractive antibacterial strategy.* Frontiers in Microbiology, 2022. **13**: p. 952633.
